# Amiodarone-Induced Electrical Storm: A Nightmare in the Emergency Room

**DOI:** 10.7759/cureus.49494

**Published:** 2023-11-27

**Authors:** Andreia Campinas, Mariana Pereira Santos, Maria João Sousa, Catarina Gomes, Severo Torres

**Affiliations:** 1 Cardiology, Centro Hospitalar Universitário de Santo António, Porto, PRT

**Keywords:** drug-induced long qt syndrome, iatrogenic event, torsa des pointes, electrical storm, side effects of amiodarone

## Abstract

Drug-induced long QT syndrome (LQTS) is defined as prolonged corrected QT interval (QTc ≥460 ms) plus polymorphic ventricular arrhythmia fitting the description of torsades de pointes temporally associated with the administration of a drug or combination of drugs. Amiodarone therapy is a known uncommon cause of acquired QT interval prolongation that should not be underestimated. We present a case of an iatrogenic electrical storm with atrial fibrillation (AF) in which amiodarone was administered to attempt chemical cardioversion, resulting in an unnoticed prolongation of the QT interval, with subsequent repeated polymorphic ventricular tachycardia, managed with isoproterenol. Concomitant drugs and slight electrolyte disturbances potentiated this phenomenon. Given the widespread use of this drug in the emergency department, our case highlights a pertinent matter for all medical emergency practitioners. Additionally, it stresses the significance of potential precipitating factors, such as electrolyte imbalances, which are clinical conditions very frequent in the emergency context, along with the importance of recognizing drug interactions. Finally, this case also emphasizes the vital importance of closely monitoring the patient's receiving amiodarone.

## Introduction

Amiodarone is one of the most widely used antiarrhythmic drugs in the emergency room because of its perceived safety and effectiveness in managing a broad range of arrhythmias, namely, for atrial fibrillation (AF) [1]. Nevertheless, it is crucial to acknowledge its potential proarrhythmic effects, which are often underestimated as it is less common compared to other antiarrhythmic drugs [2-4]. Although rare, amiodarone-induced QT prolongation can occur and degenerate into polymorphic ventricular tachycardia, also known as torsade de points, a potentially lethal complication [1-3,5,6].

## Case presentation

**Figure 1:**
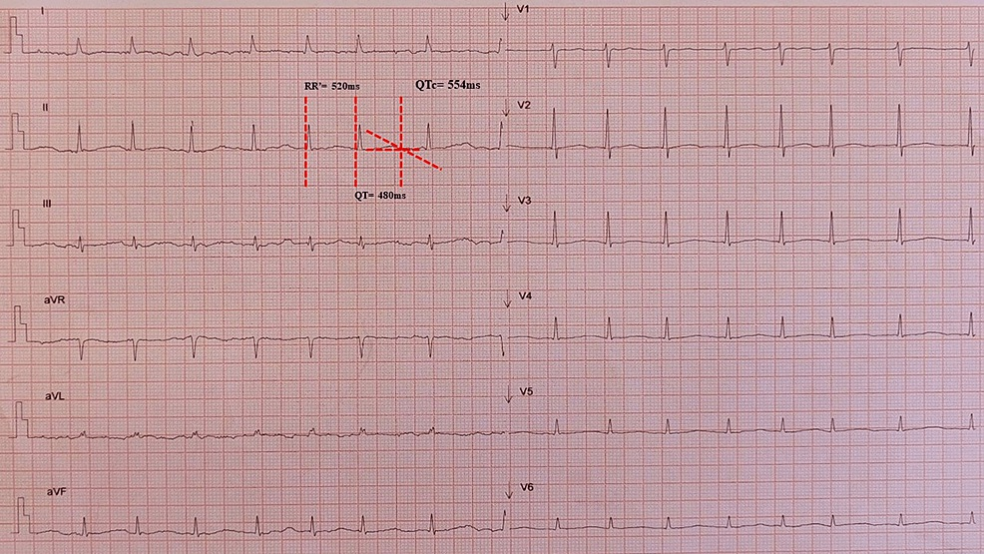
EKG performed between episodes of non-sustained polymorphic ventricular tachycardia. Atrial fibrillation with controlled ventricular rate, flattened T waves, and prolonged corrected QT interval (QTc) of 554 ms.

A 49-year-old male with alcoholic cirrhosis Child-Pugh B under tiapride (100 mg/day) and oxazepam (15 mg/day) for alcohol withdrawal syndrome was admitted to the emergency department in hemorrhagic shock because of bleeding from esophageal varices. Endoscopic treatment was performed, and an infusion of proton pump inhibitors (PPIs) was initiated. Moreover, blood transfusion and fluid therapy were provided for volume replacement (hemoglobin of 6 g/dL). Because of periods of rapid new-onset AF, intravenous amiodarone was started. He then presented with frequent non-sustained polymorphic ventricular tachycardia (Figure 1). An infusion of magnesium was administered, and the amiodarone was maintained (total dose of 2,100 mg in 36 h).

**Figure 2:**
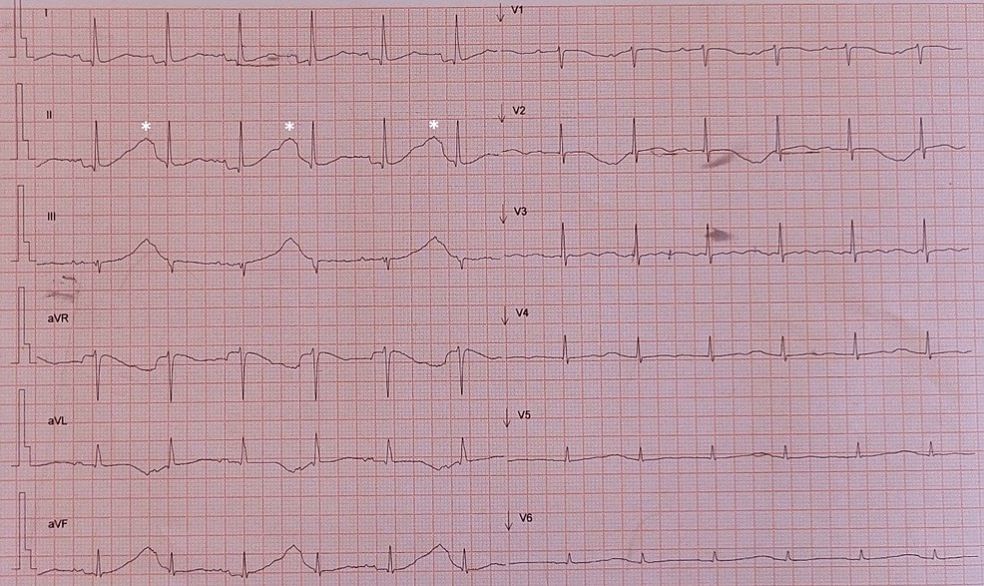
EKG performed between episodes of sustained polymorphic ventricular tachycardia. Sinus rhythm, note the extreme QTc interval prolongation (more than 700 ms) and the presence of T-wave alternans (white stars).

Given the presence of AF and repolarization changes (T-wave flattening), a significant QTc prolongation was not noticed (Figure 1). Later, he developed recurrent sustained polymorphic ventricular tachycardia with cardiac arrest requiring defibrillation several times. At this point, his EKG showed sinus rhythm and revealed a massive QTc prolongation with >700 ms with T-wave alternans (Figure 2). Blood tests (see Table 1) disclosed normal renal function with mild hypokalemia (K+ 3.2 mmol/L) and hypomagnesemia (Mg2+ 0.9 mmol/L). After multiple defibrillations, the transthoracic echocardiography (TTE) exhibited moderate global systolic dysfunction of the left ventricle.

**Table 1:** Blood analysis results.

	Result	Reference range
Creatinine mg/dL	0.9	0.70-1.20
Urea mg/dL	50	10-50
Potassium mmol/L	3.2	3.50-5.30
Magnesium mmol/L	0.7	0.74-1.07

**Figure 3:**
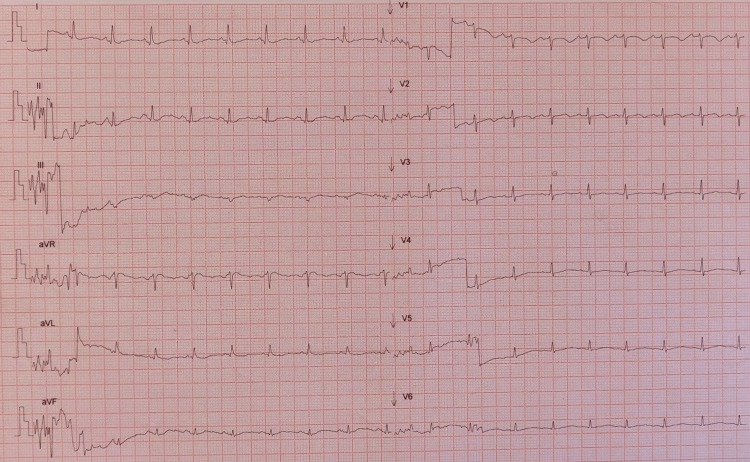
EKG showing improved QTc. Corrected QTc.

Iatrogenic QTc prolongation because of the use of amiodarone and tiapride with concomitant electrolytic disturbances was assumed. Amiodarone was stopped, electrolytic disturbances were promptly corrected, and isoproterenol with a target heart rate of 100 bpm was initiated. Ventricular dysrhythmia terminated, and QTc progressively normalized (Figure 3).

The patient was admitted to the intensive care unit for monitoring and further stabilization. Three days later, the TTE was repeated, which showed preserved left ventricular systolic function. However, a few days later, he developed severe aspiration pneumonia complicated by septic shock and died after ineffective resuscitation measures with broad-spectrum antibiotics and fluid therapy.

## Discussion

Long QT syndrome (LQTS) is characterized by the prolongation of the QT interval and by the occurrence of life-threatening tachyarrhythmias. It can be congenital or acquired [5-8]. According to the current guidelines, it is defined as a QTc ≥480 ms or a Shwartz risk score ≥3 for clinical diagnosis. In the presence of arrhythmic syncope or cardiac arrest, a QTc ≥460 ms is sufficient to consider a diagnosis of LQTS, as it is the presence of a pathogenic mutation in the genes associated with LQTS in asymptomatic patients with a normal QTc. The Schwartz score includes electrocardiographic findings (e.g., duration of the QTc interval, torsade de points, T-wave alternants, notched T-wave in three leads, and a low heart rate for age), clinical history (syncope or congenital deafness), and familial history [5,6]. Our patient met the criteria for LQTS owing to the QTc interval duration, the presence of T-wave alternants (white stars), and episodes of torsade de points.

Amiodarone is a class III antiarrhythmic agent effective in treating atrial and ventricular arrhythmias, which benefits patients with known or suspected structural heart disease [5,6]. It acts mainly by blocking the potassium channels, resulting in the prolongation of myocardial repolarization, represented by prolongation of the QT interval, particularly in the setting of predisposing conditions such as left ventricular hypertrophy, bradycardia, hypokalemia, and hypomagnesemia [2,3]. Although rare, amiodarone-induced polymorphic ventricular tachycardia is a potentially fatal complication [1-4,7-9]. Therefore, we hypothesized that the use of intravenous amiodarone and the presence of multiple precipitating risk factors (e.g., hypokalaemia and hypomagnesemia) put our patient at increased risk of developing prolonged QT and, subsequently, potentially life-threatening arrhythmic storm.

Despite the need to monitor the QTc interval, a careful analysis in the emergency department is often tricky. Additionally, the QT interval measured on the EKG varies with the RR interval, making the calculation of the QTc in AF challenging [10]. In our case, AF plus repolarization changes hindered the recognition of a significant prolongation of the QTc interval.

Tiapride, an atypical neuroleptic agent, is a selective dopamine D2-receptor antagonist. It shares the ability as the amiodarone to block the rectifier potassium channel (Ikr), resulting in delayed repolarization of the cardiac action potential and prolongation of the QT interval. Thus, the coadministration of amiodarone and tiapride may also have contributed to prolonged QT and further development of an electrical storm [11].

Traditionally, the treatment for an acquired LQTS syndrome is to correct the cause, and in some cases, transvenous pacing can be used [5,12]. Notably, current European guidelines included isoproterenol for managing acquired LQTS and recurrent dysrhythmias despite the correction of precipitating conditions [5]. It is known that amiodarone exerts its electrophysiologic effects by prolonging the action potential duration in cardiac cells (causing Ikr block) and, consequently, lengthening the effective refractory period and repolarization, manifesting as a prolongation of the QT interval on the surface EKG. Isoproterenol induces adrenergic stimulation, increasing the expression of IKs, which shortens the QTc interval [12-14]. It is a less-invasive alternative to temporary pacing overdrive in the prevention of ventricular dysrhythmias [5,12-14].

## Conclusions

Thus, our clinical case, underscored by its unique complexity and nuanced challenges, serves as a poignant reminder to a wide range of medical practitioners across diverse specialties, urging them to remain vigilant, adaptive, and collaborative in their approach to patient care.
